# Metabolic hormones and breast cancer risk among Mexican American Women in the Mano a Mano Cohort Study

**DOI:** 10.1038/s41598-019-46429-9

**Published:** 2019-07-10

**Authors:** Jie Shen, Daphne Hernandez, Yuanqing Ye, Xifeng Wu, Wong-Ho Chow, Hua Zhao

**Affiliations:** 1 Department of Epidemiology, The University of Texas MD Anderson Cancer Center, Houston, Texas, USA; 2 Department of Health and Human Performance, University of Houston, Texas, USA

**Keywords:** Breast cancer, Risk factors

## Abstract

C-peptide, insulin, leptin, and other metabolic hormones are assumed to play roles in breast cancer development; though, results are inconsistent. In this prospective case-control study nested within the Mano a Mano Cohort Study, we assessed the risk of breast cancer with regard to plasma levels of c-peptide, gastric inhibitory polypeptide, insulin, leptin, monocyte chemoattractant protein-1, pancreatic polypeptide, and peptide YY. Among women followed for a median of 8.5 years, 109 breast cancer cases were identified and frequency-matched to 327 controls at a ratio of 1:3. Overall, only c-peptide was observed significantly associated with breast cancer risk. High c-peptide levels (≥ the median level of controls) were significantly associated with increased breast cancer risk (odds ratio [OR] = 1.39, 95% confidence interval [CI]: 1.01, 2.44). In an analysis of participants stratified by age, the significant association between c-peptide levels and breast cancer risk was evident in only women age ≥51 years (OR = 1.53, 95% CI: 1.02, 3.27). Among women age <51 years, high leptin levels were significantly associated with decreased breast cancer risk (OR = 0.49, 95% CI: 0.24, 0.82). Our findings suggest that selected metabolic hormones are associated with breast cancer development in Mexican American women.

## Introduction

Metabolic syndrome and its individual metabolic conditions, including increased blood pressure, high blood sugar, excess body fat around the waist, and abnormal cholesterol or triglyceride levels, are associated with increased risk of multiple chronic diseases, including cardiovascular disease and cancer^1^. Metabolic disorders are related to every step of breast carcinogenesis^2–7^. For example, in the National Institutes of Health–American Association of Retired Persons Diet and Health Study, Dibaba *et al*. found that women with metabolic syndrome had a 13% higher risk of breast cancer compared with their female counterparts (hazard ratio [HR]: 1.13, 95% confidence interval [CI]: 1.00, 1.27)^3^. In addition, overwhelming evidence indicates that an excess of body fat is an independent risk factor for breast cancer, particularly among postmenopausal women^8,9^. Also, one recent meta-analysis of 28 cohort studies revealed that diabetic patients have a significantly higher risk of breast cancer than nondiabetic patients do(standard rate ratio = 1.13, 95% CI: 1.04, 1.24)^10^.

However, the ways in which metabolic syndrome and its individual metabolic conditions link to breast carcinogenesis is still largely undecided. The relationship may be partially mediated by certain metabolic hormones secreted by endocrine system organs, adipocytes, and/or the gastrointestinal tract in response to metabolic stimuli, as some of these hormones are suspected to play roles in breast cancer development. In the current study, we focused on 7 markers: pancreas-derived c-peptide, insulin, and pancreatic polypeptide (PP); gut-derived gastric inhibitory polypeptide (GIP) and peptide YY (PYY); and adipocyte-derived leptin and monocyte chemoattractant protein-1 (MCP-1). Experimental evidence suggests that insulin can promote breast cell growth *in vitro* and *in vivo*^11,12^. In addition, insulin deters sex hormone binding globulin production^13^, thereby increasing free estradiol and testosterone. C-peptide is a marker of pancreatic insulin secretion^14^. Leptin promotes breast cancer cell growth by hindering pro-apoptosis signaling pathways and by preferring sensitivity to estrogens^15^. PYY and PP, members of the neuropeptide Y family of peptide hormones, are neurotransmitters; both have roles in appetite regulation and obesity^16,17^. In breast tumors, neuropeptide Y can induce tumor cell growth in a dose-dependent manner^18^. GIP is an incretin hormone that is involved in regulating circulating glucose and insulin secretion^19^. Its relationship with breast cancer is unclear, but the GIP receptor has been identified as a therapeutic target in patients with neuroendocrine tumors^20^. MCP-1, a key pro-inflammatory chemokine that regulates monocyte activity, is involved in various diseases, including cancer^21^. Recently, MCP-1 was found to be highly expressed in triple-negative breast cancers and consequently involved in tumor invasion and metastasis^22^.

Investigation of metabolic hormones and their relationships with breast cancer are particularly relevant to Mexican American women, who are experiencing an epidemic of metabolic disorders^23^. More Mexican American men have elevated fasting glucose levels than non-Hispanic white men do, and more Mexican American women have high waist circumference, reduced high-density lipoprotein cholesterol, and elevated fasting glucose than their non-Hispanic white counterparts do^23^. To the best of our knowledge, no prospective study has assessed the extent to which metabolic hormone levels are associated with breast cancer risk in Mexican American women. Therefore, in the present study, we measured pre-diagnostic levels of 7 metabolic hormones in plasma samples from 109 breast cancer patients and 327 healthy controls identified from the Mano a Mano Cohort Study and investigated their relationships with breast cancer risk.

## Methods

### Study population

The study participants were drawn from the ongoing Mano a Mano Cohort Study, a large population-based prospective cohort study of Mexican American households that was initiated in 2001 by the Department of Epidemiology at The University of Texas MD Anderson Cancer Center. Eligible participants in the Mano a Mano Cohort Study had to self-identify as Mexican or Mexican American. Detailed descriptions of the recruitment strategy and data collection procedures have been described previously^24^. In brief, participants of the Mano a Mano Cohort Study were recruited through community centers, local health clinics, and house-by-house canvasing in predominantly Mexican American neighborhoods in Houston, Texas, and through networking with currently enrolled participants. Eighty-eight percent of the identified eligible households agreed to participate in the study, and written informed consent was obtained from each participant. Trained bilingual research interviewers conducted structured, face-to-face interviews using each participant’s preferred language (either Spanish or English). A standardized and validated questionnaire, which captured information on basic sociodemographic characteristics, residential history, lifestyle behaviors, physical activity, medical history, family history of chronic disease, acculturation, and occupational exposure, was used in the interview. Participants were followed with annual telephone calls to obtain updated information regarding body weight, selected exposures, and diagnosis of selected chronic diseases, including cancer, type 2 diabetes, and hypertension. The cancer cases were further confirmed with the Texas Cancer Registry.

The women were followed until December 1, 2017 (median, 8.2 years). A total of 126 new breast cancers were identified. Among them, 109 were validated through the Texas Cancer Registry and had blood samples that were collected at baseline. For each case, 3 matched controls were selected using an incidence density sampling protocol from appropriate risk sets consisting of cohort members who were alive and free of cancer at the time of diagnosis of the index case. Matching criteria were age at recruitment (±2 years), date of biospecimen collection (±1 year), and gender. Thus, the study included 109 cases and 327 controls. The study protocol was approved by MD Anderson’s Institutional Review Board.

### Quantification of metabolic hormones in plasma samples by magnetic bead-based immunoassay

Plasma samples were analyzed using Luminex multiplex technology, which assesses multiple analytes in a single microwell plate, with the MILLIPLEX MAP Human Metabolic Hormone Magnetic Bead Panel (Millipore). This panel enabled the simultaneous analysis of the 7 metabolic hormones of interest: c-peptide, GIP, insulin, leptin, MCP-1, PP, and PYY. The MAGPIX System and an xPONENT 4.2 MAGPIX analyzer (Luminex) were used to analyze the samples. All samples were analyzed in triplicate. Negative controls, standards, and positive controls were included in each plate. Blinded duplicates (5%) were randomly inserted with the samples for quality control purposes. Samples from each case and its 3 matched controls were analyzed in the same plate. Any samples yielding results with an intra-assay coefficient of variation >10% were reanalyzed. The data were processed with xPONENT software using 5-parametric curve fitting and converted to pg/mL. The intra-assay variance was 7% for c-peptide, 5% for GIP, 5% for insulin, 4% for leptin, 8% for MCP-1, 4% for PP, and 6% for PYY.

### Statistical analysis

We used the statistical software package SAS version 9.4 (SAS, Cary, NC) for all analyses. First, we evaluated whether selected sociodemographic characteristics and lifestyle behaviors differed between breast cancer patients and healthy controls; the Student t test was used for 2-level dichotomous variables, and analysis of variance was used for variables with more than 2 levels. To assess relationships among metabolic hormones, we evaluated the pairwise correlations between all hormones among the controls. Hormones with pairwise correlations >0.5 were considered to be highly correlated and to have possible redundancy. Next, we used the Wilcoxon rank-sum test to evaluate whether the median plasma levels of metabolic hormones differed according to selected sociodemographic characteristics and lifestyle behaviors of the controls. To control for multiple comparisons, we set the false discovery rate at 0.05^25^. To assess the effect of plasma levels of metabolic hormones on breast cancer risk, we used unconditional multivariate logistic regression to estimate odds ratios (ORs) and 95% CIs. We ran a minimally adjusted model, adjusting for basic demographic variables (e.g. birthplace, language acculturation, age, parity, body mass index (BMI) category, and education level), and a fully-adjusted model, with the following additional healthy behavior related variables (e.g. smoking status, drinking status, sitting time, and physical activity). Metabolic hormone levels were designated “high” or “low” using the controls’ median levels of the hormones as cutoffs and were assessed as categorical variables. Finally, in an analysis in which participants were stratified by age group, we used similar multivariate logistic regression analysis to assess relationships between metabolic hormones and breast cancer risk.

### Ethics approval

All procedures in this study were approved by MD Anderson’s Institutional Review Board and performed in accordance with the Declaration of Helsinki.

### Informed consent

Written informed consent was obtained from all participants.

## Results

The basic sociodemographic characteristics and lifestyle behaviors of the 109 breast cancer cases and 327 healthy controls are summarized in Table 1. In general, the cases and controls were well-matched. Cases and controls did not differ significantly in terms of age group, parity, education level, birthplace, language acculturation, BMI category, smoking status, alcohol drinking, physical activity, or sitting time.

**Table 1:** Distribution of characteristics among participants by case control status.

Variable	Controls, n (%)	Cases, n (%)	*P* value
Overall	327 (100)	109 (100)	
Age at enrollment, years			
<51 years	163 (49.85)	54 (49.54)	
≥51 years	164 (50.15)	55 (50.46)	0.956
Parity			
Nulliparous	28 (8.56)	9 (8.26)	
1 or 2 children	129 (39.45)	42 (38.53)	
>2 children	170 (51.99)	58 (53.21)	0.975
Education level			
<High school	206 (63.00)	70 (64.22)	
High school	64 (19.57)	20 (18.35)	
>High school	57 (17.43)	19 (17.43)	0.959
Place of birth			
Mexico	210 (64.22)	69 (63.30)	
United States	117 (35.78)	40 (36.70)	0.908
Language acculturation			
Low	209 (63.91)	61 (55.96)	
High	118 (36.09)	48 (44.04)	0.139
BMI category			
Underweight/normal weight	47 (14.37)	13 (11.93)	
Overweight	108 (33.03)	37 (33.94)	
Obese	172 (52.60)	59 (54.13)	0.814
Smoking status			
Never	238 (72.78)	70 (64.22)	
Former	69 (21.10)	28 (25.69)	
Current	20 (6.11)	11 (10.09)	0.179
Alcohol drinking			
Never	203 (62.08)	66 (60.55)	
Former	64 (19.57)	25 (22.93)	
Current	60 (18.35)	18 (16.51)	0.727
Physical activity			
Low	216 (66.06)	79 (72.48)	
Medium or high	111 (33.94)	30 (27.52)	0.215
Sitting hours per day			
<2	81 (24.77)	24 (22.02)	
2–4	84 (25.69)	36 (33.03)	
4–6	93 (28.44)	29 (26.61)	
>6	71 (21.71)	20 (18.35)	0.493

We investigated the pairwise correlations among the 7 plasma metabolic hormones in the controls and found several significant correlations (Table 2). C-peptide, GIP, insulin, and MCP-1 were significantly correlated with all other markers (P < 0.05). In particular, both c-peptide and GIP were significantly correlated with the other 6 markers (P < 0.001). C-peptide and GIP were most strongly correlated (ρ = 0.494, P < 0.001). However, no hormones had pairwise correlations >0.5, indicating that none of the hormones were highly correlated or had possible redundancy. In addition, no significant correlation was observed between leptin and PP or between leptin and PYY. After adjustment for multiple comparisons, the significant associations remained (P < 0.05), except for those between MCP-1 and insulin and between MCP-1 and PP.

**Table 2:** Pairwise correlations between plasma hormone biomarkers among controls.

	C-peptide	GIP	Insulin	Leptin	MCP-1	PP	PYY
c-peptide		0.494^1,4^	0.431^1,4^	0.357^1,4^	0.128^1,4^	0.265^1,4^	0.183^1,4^
GIP			0.454^1,4^	0.131^1,4^	0.185^1,4^	0.373^1,4^	0.332^1,4^
Insulin				0.368^1,4^	0.058^3^	0.277^1,4^	0.293^1,4^
Leptin					0.139^1,4^	−0.013	0.053
MCP-1						0.088^2^	0.176^1,4^
PP							0.272^1,4^
PYY							

GIP, gut-derived gastric inhibitory polypeptide; MCP-1, monocyte chemoattractant protein-1; PP, pancreatic polypeptide; PYY, peptide YY.

^1^P < 0.001; ^2^0.001 ≤ P < 0.01; ^3^0.01 ≤ P < 0.05; ^4^P < 0.05 after adjustment for multiple comparisons.

We assessed relationships between the plasma levels of 7 metabolic hormones and sociodemographic characteristics and lifestyle behaviors among controls (Table 3). As expected, levels of those 7 metabolic hormones increased significantly with increasing BMI category (P < 0.05). After adjustment for multiple comparisons, c-peptide, GIP, insulin, leptin, and MCP-1 levels remained significantly associated with BMI category. Plasma levels of c-peptide, GIP, insulin, MCP-1, and PYY increased significantly with increasing number of sitting hours per day (P < 0.05); even after adjustment for multiple comparisons, c-peptide and insulin levels remained significantly associated with number of sitting hours. In addition, plasma levels of c-peptide, GIP, and leptin were higher in women born in the United States than in women born in Mexico, and plasma levels of c-peptide, GIP, leptin, and MCP-1 were significantly higher in women with low levels of physical activity than in women with medium or high levels of physical activity; however, these significant associations disappeared after adjustment for multiple comparisons.

**Table 3:** Metabolic hormone levels among controls.

Variable	Median c-peptide level, pg/mL	Median GIP level, pg/mL	Median insulin level, pg/mL	Median leptin level, pg/mL	Median MCP-1 level, pg/mL	Median PP level, pg/mL	Median PYY level, pg/mL
Overall	5,164.5 (120, 9,548)	2191.5 (17, 8,877)	390 (192, 2,862)	3675.5 (133, 12,972)	1,976 (24, 7,560)	446 (16, 684)	230 (155, 1,340)
Age at enrollment							
<51 years	4,672	1,990	365	3,352	1,870	431	195
≥51 years	5,380	2,289	429	3,896	2,064	462	296
*P value*	0.368	0.763	0.378	**0**.**042**	0.734	0.523	0.631
Parity							
Nulliparous	4,768	1,973	406	3,529	1,762	426	193
1 or 2 children	4,980	2,048	372	3,796	1,965	470	240
>2 children	5,432	2,299	416	3,602	2,037	431	226
*P for trend*	0.272	0.642	0.792	0.826	0.243	0.782	0.843
Education level							
<High school	4,971	2,065	411	3,580	1,821	465	205
High school	5,306	2,090	369	3,629	1,879	429	224
>High school	5,025	2,273	392	3,781	2,165	449	239
*P for trend*	0.872	0.924	0.871	0.762	0.303	0.606	0.682
Place of birth							
Mexico	4,670	1,856	423	3,429	1,772	429	201
United States	5,608	2,437	372	3,906	2,206	458	267
*P value*	**0**.**023**	**0**.**035**	0.392	**0**.**043**	0.085	0.682	0.267
Language acculturation							
Low	4,827	2,058	365	3,629	1,987	440	224
High	5,249	2,199	479	3,702	1,923	466	262
*P value*	0.481	0.874	0.562	0.924	0.961	0.796	0.740
BMI category							
Underweight/normal	4,556	1,754	329	3,258	1,592	423	201
Overweight	5,072	2,075	387	3,541	1,824	431	219
Obese	5,591	2,396	463	3,906	2,435	469	260
*P for trend*	**<0**.**001**^1^	**<0**.**001**^1^	**<0**.**001**^1^	**<0**.**001**^1^	**<0**.**001**^1^	**0**.**026**	**0**.**018**
Smoking status							
Never	4,892	2,082	368	3,402	1,905	440	223
Former	5,079	2,187	405	3,706	1,843	453	229
Current	5,248	2,269	389	3,658	2,089	459	242
*P for trend*	0.871	0.894	0.764	0.862	0.726	0.823	0.876
Alcohol drinking							
Never	4,979	1,996	382	3,529	1,869	409	211
Former	5,105	2,257	409	3,685	1,825	460	230
Current	5,007	2,092	416	3,699	2,192	478	248
*P for trend*	0.912	0.854	0.871	0.936	0.465	0.432	0.487
Physical activity							
Low	5,409	1,898	372	3,426	1,782	413	219
Medium or high	4,678	2,367	416	3,841	2,354	460	247
*P value*	**0**.**012**	**0**.**021**	0.254	**0**.**019**	**0**.**011**	0.259	0.305
Sitting hours/day							
<2	4,580	1,846	350	3,453	1,832	413	209
2–4	4,792	1,996	369	3,826	1,945	449	226
4–6	5,081	2,357	392	3,529	2,306	438	254
>6	5,434	2,197	413	3,624	2,019	452	243
*P for trend*	**P < 0**.**001**^1^	**0**.**031**	**<0**.**001**^1^	0.258	**0**.**027**	0.165	**0**.**041**

GIP, gut-derived gastric inhibitory polypeptide; MCP-1, monocyte chemoattractant protein-1; PP, pancreatic polypeptide; PYY, peptide YY; BMI, body mass index. ^1^Statistically significant after the adjustment of multiple comparison.

We evaluated the associations between plasma levels of metabolic hormones and breast cancer risk (Table 4). For each metabolic hormone, we stratified the study participants into 2 groups based on the median plasma levels of the hormone. We included birth place, language acculturation, age, parity, BMI category, and education level in Model 1, and birth place, language acculturation, age, parity, BMI category, education level, smoking status, drinking status, sitting time, and physical activity in Model 2. C-peptide was the only metabolic hormone significantly associated with breast cancer risk. Compared with women with low c-peptide levels (<5164.5 pg/mL), women with high c-peptide levels (≥5164.5 pg/mL) had a 1.42-fold higher risk of breast cancer in model 1 (OR = 1.42, 95% CI: 1.02, 2.37) and a 1.39-fold higher risk in model 2 (OR = 1.39, 95% CI: 1.01, 2.44).

**Table 4:** Risk for developing breast cancer in relation to median levels of plasma hormone biomarkers.

Hormone	Cases	Controls	Model 1^1^	Model 2^2^
C-peptide				
<5164.5 pg/mL	44 (40.37)	163 (49.85)	1.00	1.00
≥5164.5 pg/mL	65 (59.63)	164 (50.15)	**1**.**42** (**1**.**02**, **2**.**37**)	**1**.**39** (**1**.**01**, **2**.**44**)
GIP				
<2191.5 pg/mL	52 (47.71)	164 (50.15)	1.00	1.00
≥2191.5 pg/mL	57 (52.29)	163 (49.85)	1.09 (0.67, 1.79)	1.06 (0.63, 1.84)
Insulin				
<390 pg/mL	50 (45.87)	162 (49.54)	1.00	1.00
≥390 pg/mL	59 (54.13)	165 (50.46)	1.12 (0.70, 1.87)	1.08 (0.64, 1.96)
Leptin				
<3675.5 pg/mL	60 (55.05)	164 (50.15)	1.00	1.00
≥3675.5 pg/mL	49 (44.95)	163 (49.85)	0.84 (0.50, 1.31)	0.85 (0.48, 1.35)
MCP-1				
<1976 pg/mL	53 (48.62)	162 (49.54)	1.00	1.00
≥1976 pg/mL	56 (51.38)	165 (50.46)	1.04 (0.53, 1.67)	1.01 (0.48, 1.75)
PP				
<446 pg/mL	54 (49.54)	163 (49.85)	1.00	1.00
≥446 pg/mL	55 (50.46)	164 (50.15)	1.02 (0.45, 1.74)	1.01 (0.43, 1.77)
PYY				
<230 pg/mL	52 (47.71)	164 (50.15)	1.00	1.00
≥230 pg/mL	57 (52.29)	163 (49.85)	1.07 (0.64, 1.80)	1.05 (0.62, 1.86)

GIP, gut-derived gastric inhibitory polypeptide; MCP-1, monocyte chemoattractant protein-1; PP, pancreatic polypeptide; PYY, peptide YY.

^1^Adjusted for birthplace, language acculturation, age, parity, body mass index category, and education level.

^2^Adjusted for birthplace, language acculturation, age, parity, body mass index category, education level, smoking status, drinking status, sitting time, and physical activity.

When study participants were further stratified using the median age of the controls as a cutoff, higher c-peptide levels were significantly associated with breast cancer risk in only women age ≥51 years after adjustment for birthplace, language acculturation, parity, BMI category, and education level (OR = 1.53, 95% CI: 1.02, 3.27) (Table 5). Interestingly, among women age <51 years, leptin levels were inversely associated with breast cancer risk (OR = 0.49, 95% CI: 0.24, 0.82). In addition, we analyzed whether the risk associations differed by obesity status (Table 5). Unfortunately, no significant association was observed between metabolic hormones and breast cancer risk in either non-obese or obese group.

**Table 5:** Risk for developing breast cancer in relation to median levels of plasma hormone biomarkers by age category.

Hormone	Age < 51 years	Age ≥ 51 years	non-obese	Obese
C-peptide				
Low	1.00	1.00	1.00	1.00
High	1.36 (0.87, 3.56)	**1**.**53** (**1**.**02**, **3**.**27**)	1.37 (0.90, 3.78)	1.46 (0.98, 3.69)
GIP				
Low	1.00	1.00	1.00	1.00
High	1.03 (0.59, 3.98)	1.08 (0.52, 4.01)	1.05 (0.60, 3.75)	1.06 (0.50, 3.86)
Insulin				
Low	1.00	1.00	1.00	1.00
High	1.06 (0.63, 3.83)	1.05 (0.59, 4.10)	1.03 (0.60, 3.79)	1.04 (0.58, 4.02)
Leptin				
Low	1.00	1.00	1.00	1.00
High	**0**.**49** (**0**.**24**, **0**.**82**)	1.39 (0.92, 3.76)	0.75 (0.43, 1.92)	1.28 (0.90, 3.81)
MCP-1				
Low	1.00	1.00	1.00	1.00
High	0.89 (0.43, 3.59)	1.23 (0.57, 4.05)	1.04 (0.41, 4.06)	0.99 (0.40, 4.12)
PP				
Low	1.00	1.00	1.00	1.00
High	0.93 (0.39, 3.77)	1.04 (0.47, 3.97)	0.98 (0.43, 3.86)	1.02 (0.49, 4.01)
PYY				
Low	1.00	1.00	1.00	1.00
High	0.99 (0.40, 3.85)	1.07 (0.51, 4.08)	0.98 (0.39, 4.01)	1.02 (0.43, 3.99)

GIP, gut-derived gastric inhibitory polypeptide; MCP-1, monocyte chemoattractant protein-1; PP, pancreatic polypeptide; PYY, peptide YY.

^1^Adjusted for birthplace, language acculturation, parity, body mass index category, and education level as appropriate.

## Discussion

To our knowledge, the current study was the first to prospectively assess associations between circulating metabolic hormone levels and breast cancer risk in Mexican American women. We found that higher c-peptide levels were significantly associated with an increased risk of breast cancer and that this risk was more evident among older women than among younger women. In addition, among younger women, higher leptin levels were significantly associated with a decreased risk of breast cancer.

Several previous studies have investigated the association between pre-diagnostic c-peptide levels and breast cancer risk^26–29^. These studies consistently showed a significant relationship between c-peptide levels and breast cancer risk in older or postmenopausal women^27,29^ but not younger or premenopausal women^26^. For example, in the European Prospective Investigation into Cancer and Nutrition study, higher serum c-peptide levels were associated with higher breast cancer risk among women age >60 years, but not among their younger counterparts^27^. In the Cancer Prevention Study II Nutrition Cohort, a significant association between higher levels of c-peptide and breast cancer risk was observed among postmenopausal women^29^. In agreement with those reports, in the current study, higher plasma levels of c-peptide were significantly associated with breast cancer risk in women age ≥51 years (OR = 1.53, 95% CI: 1.02, 3.27) but not women age <51 years. In addition, when younger and older women combined together, the association remained significant. Although the observed age difference in risk association is consistent the literature reports, we cannot exclude the likelihood of probable biases/measurement error that can influence the observation. Our study used BMI to identify obesity. However, a recent study has shown that BMI is a suboptimal marker for adiposity in the elderly^30^. Also, there may exist detection bias since tumor may be diagnosed later among obese women.

C-peptide, which is released into the blood as a byproduct of insulin, is considered to be a marker of insulin production and hyperinsulinemia^31^. Hyperinsulinemia with insulin resistance, which causes increased levels of insulin in circulation, has been linked to breast cancer^32–34^. Thus, the association of higher plasma levels of c-peptide with elevated risk of postmenopausal breast cancer is consistent with the notion that hyperinsulinemia is involved in in breast cancer development. Two hypotheses have been proposed to explore the underlying molecular mechanism. C-peptide may 1) potentiate the insulin receptor and/or 2) increase the concentration of bioavailable sex hormones, and thereby influence the action of insulin on breast cancer cell growth directly and/or indirectly^35^. Additionally, stimulation of the mitogen-activated protein kinase (MAPK) and the phosphoinositide 3-kinase (PI-3K) pathways has been proposed to be involved in the action of insulin and its receptor on promoting cell growth^35,36^.

In the present study, plasma c-peptide levels were significantly positively associated with BMI and BMI category. This finding is in line with the notion that obesity causes insulin resistance and hyperinsulinemia. This observation might also help explain the observed age difference in the association between c-peptide and breast cancer risk, as BMI is a protective factor for breast cancer in premenopausal women but a risk factor for breast cancer in postmenopausal women. Nevertheless, the observed risk continued fairly unchanged by adjustments for BMI, signifying that the influence of insulin on breast cancer risk was not related to excess weight.

In our study, the relationship between leptin and breast cancer risk differed depending on age; increased plasma leptin levels were significantly associated with decreased breast cancer risk in younger participants (<51 years old) but not older participants (≥51 years old). This finding is in line with the results of several other cohort studies^37,38^. For example, in a prospective case-control study nested within the Nurses’ Health Study II cohort, after adjusting for BMI at age 18 years, weight change from age 18 years to blood draw, and other breast cancer risk factors, plasma leptin was a protective factor for breast cancer^37^. Leptin is thought to be a link between obesity and obesity-related complications including metabolic syndrome, type 2 diabetes, and cancer^39^. In breast epithelial cells, leptin can stimulate cell proliferation in obese women by accelerating the change of aromatizable androgens to estradiol. However, among postmenopausal women, their levels of circulating estrogens decline^40,41^. That is probably why we didn’t see significant association between leptin and breast cancer risk in older women. On the other hand, leptin is involved in the regulation of ovarian folliculogenesis^42^ and at high levels may reduce follicular estradiol secretion^43^. Such reduction is particularly relevant to premenopausal or younger women since it can help explain why high leptin may lower breast cancer risk among them.

In the present study, GIP, insulin, leptin, and MCP-1, in addition to c-peptide, were significantly associated with BMI, even after adjustment for multiple comparisons. This finding is consistent with these hormones’ roles in metabolic syndrome and its individual metabolic conditions. We also found that levels of c-peptide and insulin were significantly increased with the time spent sitting per day after multiple comparison adjustment. This is consistent with the observation that being less physically active is a risk factor for insulin resistance^44^.

Our study had several potential limitations. For example, we measured metabolic markers at only one time point, which prevented us from evaluating the value changes over time. Data on menopausal status at the time of diagnosis were lacking. Hence, we chose to use age at diagnosis as an estimation of menopausal status at the time of diagnosis when stratifying participants. In addition, the blood samples used in this study were collected from patients who had not been fasting. Fasting plasma metabolic markers may be better biomarkers than non-fasting ones^27^. Finally, we don’t have data on estrogen status so we cannot assess whether the relationships between plasma metabolic hormones and breast cancer risk differ by tumor subtype. A few studies have suggested that the relationship between obesity and breast cancer risk differs between estrogen receptor positive (ER+) and ER− breast tumors in postmenopausal women^45–47^. Given the strong correlations between those metabolic hormones and obesity, it is likely that the observed associations between plasma metabolic hormones and breast cancer risk are more evident among for ER+ than ER- breast tumors. However, in an analysis from Nurse Health Study, the association between plasma c-peptide and breast cancer was stronger among ER− than ER+ breast tumors^48^. Clearly, more studies at here filed are needed.

## Conclusions

Despite these potential limitations, our results provide the first evidence that higher c-peptide levels are significantly associated with increased breast cancer risk among older Mexican American women and that higher leptin levels are significantly associated with decreased risk of breast cancer among younger Mexican American women. Large prospective studies to validate our results are warranted.
